# Arbuscular mycorrhizal fungi regulate mineral element distribution in grapevines under pH stress

**DOI:** 10.3389/fpls.2026.1785602

**Published:** 2026-04-07

**Authors:** Dehui Sun, Wensheng Du, Yaru Wang, Yangyong Wang, Shengji Wei, Jiakun Qin, Zhen Gao, Qinghua Sun, Yuanpeng Du

**Affiliations:** 1 College of Horticulture Science and Engineering Shandong Agricultural University, Tai’an, Shandong, China; 2 College of Mechanical Engineering, Taishan University, Tai’an, Shandong, China

**Keywords:** grapevines, growth and development, mineral elements, pH, *Septoglomus viscosum* and *Glomus chinensis*

## Abstract

Grapevine (*Vitis vinifera* L.) productivity is closely linked to soil stability, and pH is a master variable controlling both plant development and the bioavailability of essential mineral elements (Mg, Ca, Fe, Cu, Zn). This study investigates the growth-promoting effects of arbuscular mycorrhizal fungi (AMF) on grapevines under different soil pH environments. The results indicate that compared to pH 6.5, the relative electrical conductivity (REC) of leaves, root malondialdehyde (MDA), and reactive oxygen species (ROS) increased at pH 5 and pH 8, affecting the absorption and transport of Mg, Ca, Fe, Cu, and Zn, thereby inhibiting the growth and development of grapevines. Inoculation with the AMF *Septoglomus viscosum* and *Glomus chinensis* significantly enhanced the activities of superoxide dismutase (SOD) and peroxidase (POD) by activating the root antioxidant system, thereby alleviating the impact of pH stress on grapevine growth and development. Under pH 8 condition, the effects were more pronounced, with *G. chinensis* significantly increasing plant fresh weight (103.06%), net photosynthetic rate (53.99%), root vitality (108.70%), ferric chelate reductase (FCR) (54.80%), and POD (49.23%), while significantly reducing leaf REC (33.33%) and root MDA content (35.79%). *S. viscosum* facilitated the root absorption and upward transport of Mg and Ca, significantly promoting the accumulation of Zn and Cu in the roots and inhibiting their transport to the above-ground parts, thereby alleviating heavy metal stress on the leaves. Overall, the addition of AMF significantly improves the distribution of Mg, Ca, Fe, Zn, and Cu within grapevines, enhancing leaf and root functions as well as biomass accumulation under acid-base stress conditions. These findings demonstrate that *S. viscosum* and *G. chinensis* differentially promote grapevine performance across pH gradients, offering mechanistic insights into pH-dependent mineral nutrient homeostasis. They provide a theoretical basis for using AMF-based biotechnologies in sustainable viticulture to enhance stress resilience and fruit quality.

## 1 Introduction

Grapevine (*Vitis vinifera* L.) is one of the world’s most economically significant perennial fruit crops, with its growth and productivity closely linked to environmental conditions. Ongoing climate change, coupled with suboptimal agricultural management practices, has exposed vineyards worldwide to increasing abiotic stresses, including soil degradation, water deficit, salinization, and heavy metal contamination. These stress factors severely restrict grapevine growth by damaging the physicochemical properties of the soil, limiting root development, and disrupting nutrient balance (Ahmadi Nouraldinvand et al., 2022; Iqbal et al., 2025; Li et al., 2025; Rouhollah et al., 2022). Among these factors, soil pH emerges as a master variable governing both soil fertility and vine health (Eleonora et al., 2021). The typical soil pH range in Chinese vineyards is between 5.0 and 8.0. A neutral pH of 6.5 to 7.0 is generally considered ideal, as it represents a balance point where mineral nutrients are optimally soluble, thereby supporting maximum grapevine growth (Li et al., 2024; Yan et al., 2022).

However, excessive application of synthetic fertilizers and organic manures, coupled with irrigation water quality and soil parent material characteristics, has exacerbated soil acidification or alkalinization in numerous grape-producing regions, pushing soil pH toward extreme values. Both extreme acidic and alkaline soil environments can disrupt root cell membrane permeability and induce oxidative damage. This, in turn, affects the solubility of metal cations in the soil and reduces the plant’s ability to absorb mineral elements, ultimately impacting stress resistance, growth, and yield stability (Nie et al., 2024; Silva et al., 2025; Vanessa et al., 2023; Ye et al., 2024). Nutrient imbalance caused by pH stress affects processes ranging from root health to ecosystem function, and has emerged as a key abiotic factor threatening the sustainable development of vineyards (Beheiry Hamada and Z., 2023; Miriam et al., 2022).

Arbuscular mycorrhizal fungi (AMF) are ubiquitous symbiotic partners in terrestrial ecosystems and are particularly effective in promoting plant phosphorus (P) uptake (Kobae, 2019; Wenda et al., 2022). In addition to enhancing P acquisition, AMF modulate the absorption of other mineral elements, such as calcium (Ca), magnesium (Mg), iron (Fe), zinc (Zn), and copper (Cu), by expanding the root exploration zone and regulating rhizosphere pH (Chandrasekaran et al., 2021; Fofana et al., 2022). The hyphal networks produced by AMF can expand the root absorption area several-fold and, by modulating the rhizosphere pH microenvironment, enhance root vigour, thereby creating conditions more favorable for root growth and nutrient uptake (Asmarahman et al., 2023; Muriithi-Muchane, 2013). This enables plants to better adapt to stressful environments and promotes overall growth and development (Nohesara et al., 2025; Paula et al., 2022; Pu et al., 2025). These regulatory effects on nutrient dynamics position AMF as a promising tool for sustainable viticulture, capable of assisting grapevines in maintaining normal physiological metabolism under adverse soil pH conditions.

However, limited information is available regarding how root functions influence the allocation of mineral elements (Ca, Mg, Fe, Cu, Zn) between shoot and root tissues of grapevines under contrasting pH environments. We hypothesize that, under varying pH conditions, AMF modulate root physiological functions—including protein metabolism and antioxidant systems—and that these modifications are critical for maintaining the balance of mineral element distribution within grapevines. Furthermore, this regulation mediated by AMF can indirectly enhance leaf function, ultimately improving grapevine tolerance to both acidic and alkaline stress. These findings provide a mechanistic understanding of how soil pH, through its effects on root physiological metabolism, influences the allocation of mineral elements (Ca, Mg, Fe, Cu, Zn) within grapevines. This knowledge offers a theoretical basis for sustainable nutrient management and the development of pH stress tolerance strategies in grape cultivation.

## 2 Materials and methods

### 2.1 Plant materials and treatments

#### 2.1.1 Source and propagation of AMF

Root soil samples were collected from grape planting areas in Heze, Binzhou, and Jining, where two AMF, *Septoglomus viscosum* and *Glomus chinensis*, were found to be commonly present. The *S. viscosum* inoculum was obtained from the Subtropical AM Fungal Resource Conservation Center, while the *G. chinensis* inoculum was sourced from the State Key Laboratory of Grassland Agricultural Ecosystems at the College of Ecology and Life Sciences.

The substrate was prepared by mixing river sand in a ratio of 18 mesh:10 mesh:4 mesh 2:1:1 (v/v/v). For each pot, 1 kg of mixed substrate was used, and 50 g of each fungal agent was applied using a layered sowing method. At 1/3 of the distance from the bottom of the pot, 25 g of the fungal agent was sprinkled, followed by a layer of river sand covering it up to 1/2 or 2/3 of the pot’s height. Another 25 g of the fungal agent was then applied, covered with a layer of river sand to conceal the fungal agent. A layer of vetiver grass seeds was then sown, followed by another layer of river sand, after which sterile water was irrigated until a small amount of water flowed out from the bottom of the pot. The pots were placed in a cultivation room for 3–4 months, during which they were irrigated with sterile water and sterile Hoagland nutrient solution 2–3 times per week, with 100 ml each time. Upon harvesting, a portion of the root system was cut using sterile scissors and observed under a microscope to check for infection. After confirming infection, in a sterile environment, the aboveground parts of the plants were removed with sterile scissors, and the remaining root system was chopped and stored in a sealed bag at 4°C in the refrigerator.

### 2.1.2 Experimental treatments

The experiment was conducted from July to October 2024 at the Grape Experimental Park of Shandong Agricultural University, Tai’an, China (117.16°E, 36.17°N). One-year-old own-rooted ‘Sunshine Rose’ grapevines were grown in plastic pots (210 mm × 190 mm). The substrate was a 2:1 (v/v) mixture of river sand and gravel, sterilized by autoclaving prior to use. The soil pH range of 6.5-7.0 was deemed suitable for normal plant growth. Based on a pH of 6.5 as the control environment, three gradients were set: pH 5 (acidic environment), pH 6.5 (normal environment), and pH 8 (alkaline environment). Hydrochloric acid and sodium hydroxide were used for pH adjustment. For each pH gradient, a control group (CK, non-inoculated) was established, along with treatments inoculated with *S. viscosum* (NZ) and *G. chinensis* (ZH). Each fungal agent was applied using a layered sowing method: 40g of the agent was sprinkled at 1/3 the height from the bottom of the pot, then covered up to half the height of the pot, followed by another 40g of the agent placed with the grape seedlings, and finally covered to 1/4 from the top of the pot. The corresponding Hoagland nutrient solution, with pH adjusted, containing iron (50 µmol/L) was irrigated, with 200 mL of sterile nutrient solution applied one to two times weekly, depending on weather conditions, to maintain the pH environment of the potted root system. Each treatment was replicated four times.

### 2.2 Sample collection

On a sunny morning in October 2024, measurements of new shoot growth, leaf area, photosynthesis, and fluorescence were conducted. In the afternoon, fresh leaf and root samples were collected from each treatment, immediately placed on ice, and transported to the laboratory. Leaves and roots were rinsed with deionized water. Subsamples were processed immediately for determination of chlorophyll, relative electrical conductivity (REC), root activity (RA), malondialdehyde (MDA), protein, ferric chelate reductase (FCR) activity, antioxidant enzymes (POD, SOD), and reactive oxygen species (ROS) in roots; remaining tissues were stored at −80 °C for subsequent analyses. For mineral element analysis, leaf and root samples were oven-dried at 80 °C for 24 h, ground to a fine powder, and used for determination of Mg, Ca, Fe, Cu, and Zn concentrations.

### 2.3 Mycorrhizal colonization identification

Spores were extracted from rhizosphere substrate by wet sieving and decanting. Briefly, 50 g of a substrate from the root zone, air dried, was mixed with sterile water and stirred. After settling for 30 min, the suspension was passed through a stack of sterile 10 mesh and 20 mesh sieves; the residue retained on the 20 mesh sieve was collected. The residue was rinsed clean and placed into a centrifuge tube, which was then centrifuged at 3000 r/min for 3 minutes. The supernatant was filtered again and collected in a Petri dish for later use. In the centrifuge tube, 45% sucrose was added for a second centrifugation (1500 r/min, 2 minutes). The first steps were repeated, and the suspension was filtered and collected in the Petri dish. Finally, the number of spores was observed under a stereomicroscope. Calculations indicated that the inoculum of *S. viscosum* contained 401 spores 10g^-^¹, while the inoculum of *G. chinensis* had 388 spores 10g^-^¹, as determined by the wet sieving and decanting method. In a sterile environment, each group selected 10 sample repetitions, with each repetition involving a 10 cm root segment. The roots were washed with sterile water and dried, then subjected to bleaching (3 mL of 40% NaOH + 30 mL of 10% H_2_O_2_) for 15 minutes, acidification (5% lactic acid) for 5 minutes, staining (5% trypan blue), and water bath treatment at 90°C for 1 hour. Decolorization was performed by soaking in sterile water for 30 minutes. Each repetition was cut into 1 cm root segments (a total of 10 groups), and specimens were prepared using 50% glycerin. The infection status was observed using a Leica microscope (×20). In total, 10×10 groups of 1 cm root segments were analyzed, and the number of infected root segments was counted. The infection frequency was calculated using the formula: infection quantity/total root segment quantity = infection frequency (Guiwei et al., 2023).

### 2.4 Plant physiological indexes

The aboveground and belowground parts of the grapevine, as well as the total fresh weight, were measured using an electronic scale (1 kg); the growth of new shoots was measured using a tape measure; and the leaf area of the grapevine leaves was determined using coordinate paper.

The leaf fluorescence sensor and SPAD instrument were used to measure fluorescence; the photosynthesis system (SYSTEMS-CIRAS-3) was employed to determine the net photosynthetic rate, intercellular carbon dioxide concentration, and stomatal conductance. The leaf chlorophyll content was determined using an ethanol extraction method with slight modifications. A sample of 0.2 g of plant tissue (avoiding the leaf veins) was washed, dried, and chopped into a 50 mL centrifuge tube, to which 10 mL of 95% ethanol was added for dark extraction over 24 hours. After 24 hours, the solution was diluted to the 25 mL mark and colorimetric measurements were taken at wavelengths of 470, 649, and 665 nm (Kang et al., 2025).

Root vitality (RA) was determined using the triphenyl tetrazolium chloride (TTC) method. A 0.1g sample was taken and mixed with 5mL of 0.4% TTC and 5mL of phosphate solution (pH=7.0), sealed, and incubated in a constant temperature chamber at 37°C for 4 hours. Afterward, 2mL of 1mol/L H2SO4 was added, allowed to stand for 15 minutes, and the solution was discarded. Then, 10mL of 95% ethanol was added, sealed, and left to stand for 24 hours before measuring absorbance at 485nm (Yufei et al., 2022).

Malondialdehyde (MDA) content in the roots was determined using the thiobarbituric acid (TBA) colorimetric method with slight modifications. The samples were ground in liquid nitrogen, and 0.5g was mixed with 5mL of 10% trichloroacetic acid (TCA). The mixture was shaken for 10 minutes and centrifuged at 4000r/min. Then, 2mL of the supernatant was mixed with 2mL of 0.6% thiobarbituric acid, heated in a boiling water bath for 15 minutes, and centrifuged again before measuring absorbance at 450, 532, and 600nm (Zhang et al., 2026).

The content of reactive oxygen species (O_2_^-^, H_2_O_2_) in the root system was determined using a phosphate buffer extraction method (pH 7.8). A sample of 1g was ground in liquid nitrogen and then mixed with 5mL of 50mM phosphate buffer (pH 7.8). The mixture was centrifuged at 12000r/min for 5 minutes at 4°C. A 1mL aliquot of the supernatant was combined with 1mL of phosphate buffer, 1mL of 17mM para-aminobenzoic acid, and 1mL of 7mM α-naphthylamine, and the color was developed at 25°C for 20 minutes. The absorbance of O_2_^-^ was measured at 530nm. Another 1mL aliquot of the supernatant was mixed with 1mL of 5% (W/V) titanium sulfate, allowed to stand for 10 minutes, then centrifuged at 12000r/min for 10 minutes at 4°C. The supernatant was used to measure H_2_O_2_ at 410nm (Lei et al., 2020; Yan et al., 2025).

The POD assay was performed as guaiacol method with little modifications.The 0.1 mL enzyme solution was mixed with 1.0 mL H_2_O_2_ (0.2 mM, pH = 7.0), 2.9 mL PBS (0.05 M, pH = 7.0), and 1.0 mL guaiacol (0.05 M). The absorbance of mixture was measured at 470 nm. One unit of POD activity was defined as an increase in absorbance of 0.1 per milligram of protein per minute.;The SOD assay was performed as nitro blue tetrazolium (NBT) method with little modifications. The 0.1 mL enzyme solution was mixed with 2.9 mL PBS (0.05 M, pH 7.0) containing 2.0 µM riboflavin, 75.0 µM NBT, 10.0 µM ethylenediaminetetraacetic acid, and 13.0 µM methionine. The mixture was irradiated by fluorescent lamp (4000 lx) for 20 min. The absorbance was measured at 560 nm. One unit of SOD activity was defined as the amount of enzyme required to inhibit the reduction of NBT by 50% per milligram of protein. The above experiments were repeated three times (Zhixiang et al., 2023).

Root proteins were extracted using the Bradford method, with slight modifications. Total protein was extracted from frozen root powder using ice cold extraction buffer (50 mM PBS pH 7.4, 1 mM EDTA, 1 mM DTT, 1 mM PMSF, and 1% protease inhibitor cocktail). After incubation on ice for 30 min, the homogenate was centrifuged at 12,000 ×g for 20 min at 4 °C, and the supernatant was collected. Protein concentration was determined by the Bradford method using bovine serum albumin (BSA) as the standard. Absorbance was measured at 595 nm (Kielkopf Clara et al., 2020; Lalit et al., 2016).

The enzyme activity of Ferric Chelate Reductase (FCR) was measured using the Chelate Reductase Activity Assay Kit, provided by Beijing Solarbio Science & Technology Co., Ltd.

### 2.4 Determination of Ca, Mg, Fe, Zn and Cu contents in aerial parts and underground parts

The concentrations of Mg, Ca, Fe, Cu, and Zn in the leaves and roots were determined using the digestion method with nitric acid and perchloric acid (Badran et al., 2014). A sample of 0.5 g was placed in a glass conical flask, and 10 mL of a mixed acid solution (perchloric acid: nitric acid = 1:4) was added. The mixture was digested on a digestion furnace at high temperature until discoloration occurred. After cooling for 8 hours, the solution was transferred to a 50 mL centrifuge tube and diluted to a final volume of 40 mL for storage at room temperature. The concentrations were measured using an atomic absorption spectrophotometer.

### 2.5 Statistical analysis

Data for each treatment were presented as the mean of four replicates with standard error. Data were statistically analyzed using SPSS 26.0. One-way ANOVA was used for comparison of means, and LSD, Tukey’s-b, and Waller-Duncan tests were used for *post hoc* multiple comparisons. Descriptive statistics and homogeneity of variance tests were performed. Differences were considered significant at p < 0.05 and extremely significant at p < 0.01.

Graphing and visualization were performed using OriginPro 2025b. The applied procedures included Paired Comparison Plot, Correlation Plot (Pearson correlation), Heat Map with Dendrogram (Single Linkage clustering method, Cosine similarity), and Principal Component Analysis (PCA) based on correlation matrix with 12 extracted components in 2D plots.

## 3 Results

### 3.1 Microscopic image of AMF colonization

**Figure 1:**
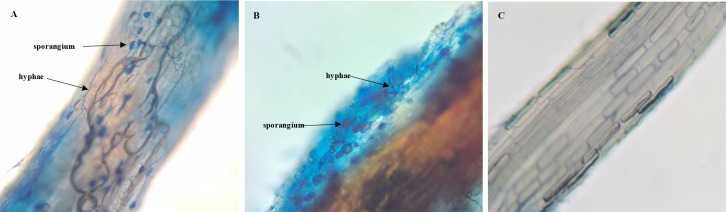
Microscopic Image of AMF Colonization. *Septoglomus viscosum* **(A)**, *Glomus chinensis* **(B)**, CK **(C)**.

Microscopic observation revealed that, compared to the CK treatment (**Figure 1C**), both the NZ treatment inoculated with *S. viscosum* (**Figure 1A**) and the ZH treatment inoculated with *G. chinensis* (**Figure 1B**) exhibited significant colonization and infection. Based on the statistical analysis of infection frequency, *S. viscosum* showed infection frequencies of 48%, 68% (**Supplementary Table 2**), and 57% at pH 5, pH 6.5, and pH 8, respectively. In contrast, *G. chinensis* demonstrated infection frequencies of 51%, 70%, and 63% (**Supplementary Table 2**) at the same pH levels. Notably, the infection frequency of *G. chinensis* was superior to that of *S. viscosum*, especially at pH 8, where the difference was most pronounced.

### 3.2 The effect of AMF on grape plant biomass across a pH gradient

**Figure 2:**
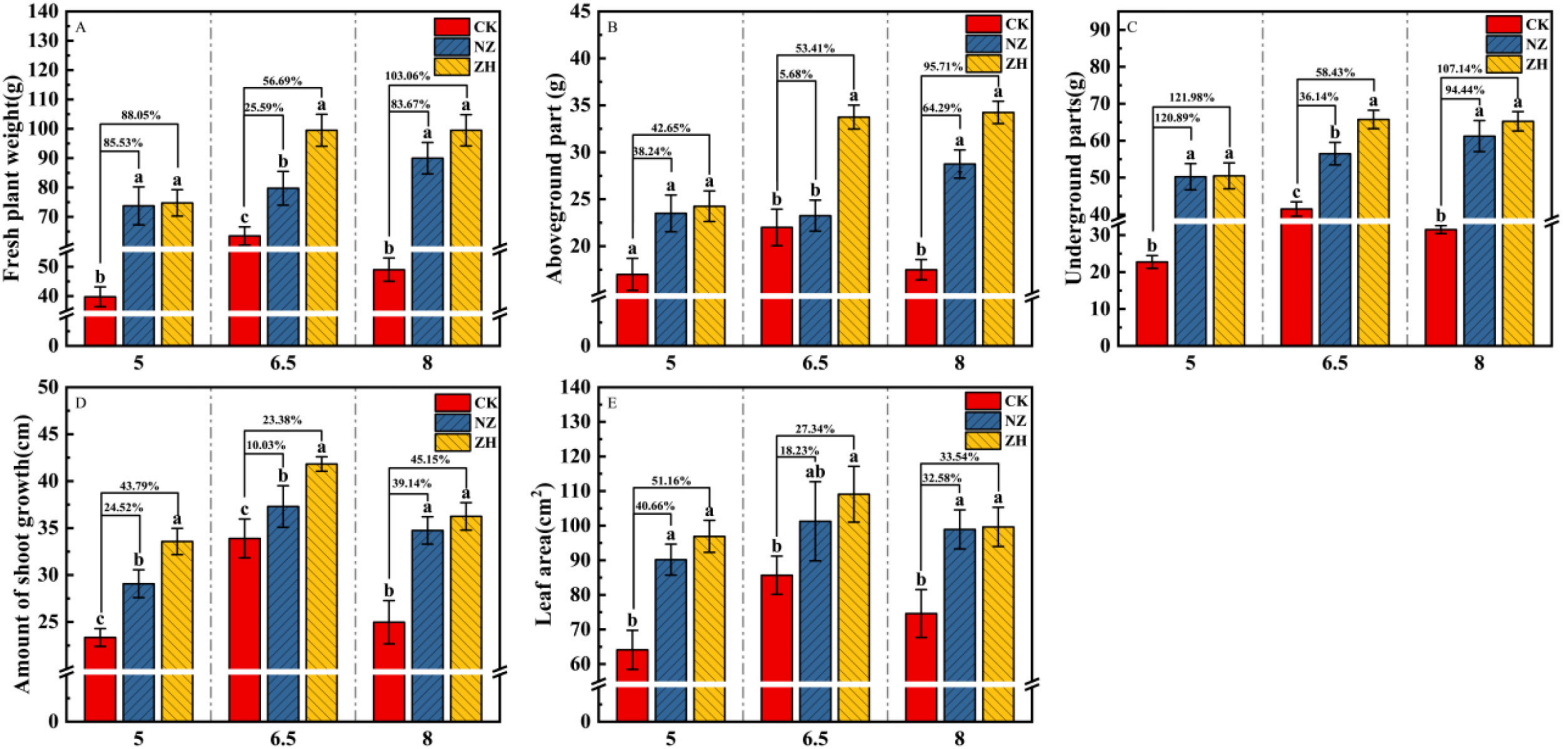
The effects of *S. viscosum* and *G. chinensis* on the biomass of grapevines were examined. The fresh weight of the plants **(A)**, fresh weight of the aboveground parts **(B)**, fresh weight of the underground parts **(C)**, Amount of shoot growth **(D)**, leaf area **(E)** were measured. The percentages indicate the enhancement compared to the CK treatment, with the NZ and ZH treatments showing respective percentage increases. Lowercase letters indicate significant differences between treatments (P < 0.05). Values shown are the mean ± standard error (n = 3).

Different pH gradients have varying effects on grapevine growth. Compared to pH 6.5, both pH 5 and pH 8 inhibit plant growth, with the inhibitory effect on grapevine biomass being greater at pH 5 than at pH 8 (**Figure 2**). AMF can effectively enhance the fresh weight of various plant organs, promote new shoot growth, and increase leaf area under different soil pH conditions, with the best performance observed in the ZH (*G. chinensis*) treatment. Under the pH 8 gradient, there was a significant increase in plant and aboveground fresh weight and new shoot growth (**Figures 2A, B, D**), which improved by 103.06%, 95.71%, and 45.15% respectively compared to the control. Under the pH 5 gradient, there was a significant increase in underground fresh weight and leaf area (**Figures 2C, E**), which improved by 121.98% and 51.16% respectively compared to the control. At pH 6.5, the growth of new shoots and leaf area was highest.

### 3.3 The impact of AMF on grape leaf physiology under varying pH gradients

**Figure 3:**
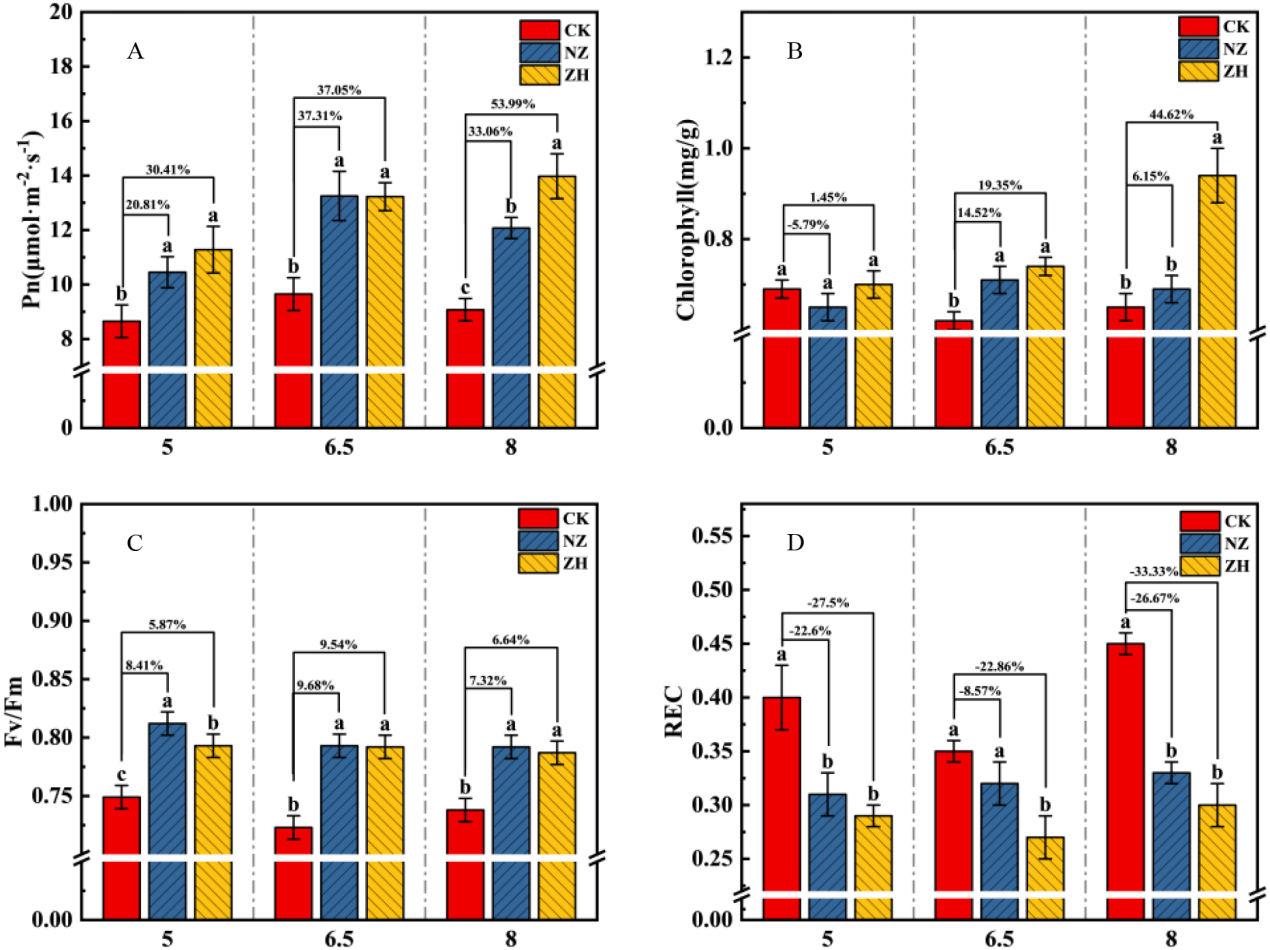
Effects of AMF on grape leaves at different pH levels: Net photosynthetic rate **(A)**, Chlorophyll **(B)**, Leaf Fluorescence **(C)**, and Relative Electrolyte Conductivity of Leaves **(D)**. *S. viscosum*(NZ) and *G. chinensis*(ZH). The percentages indicate the enhancement compared to the CK treatment, with the NZ and ZH treatments showing respective percentage increases. Lowercase letters indicate significant differences between treatments (P < 0.05). Values shown are the mean ± standard error (n = 3).

Different pH conditions significantly affect the physiological state of grapevine leaves. Compared to pH 6.5, both pH 5 and pH 8 significantly increased the relative electrolyte conductivity (REC) and suppressed the net photosynthetic rate of the leaves (**Figure 3**). The addition of AMF plays a positive role in helping plants adapt to acidic and alkaline stress environments, improving leaf functions, enhancing the net photosynthetic rate, chlorophyll content, and fluorescence, while significantly reducing REC (**Figure 3**). The ZH treatment exhibited the best overall performance, particularly at pH 8, where the net photosynthetic rate, chlorophyll content, and stomatal conductance (gs) (**Figures 3A, B**, **S1F**) increased by 53.99%, 44.62%, and 57.96% respectively, while REC significantly decreased by 33.33% (**Figure 3D**). The NZ treatment significantly enhanced the fluorescence of grapevine leaves, with the leaf fluorescence and intercellular CO_2_ concentration (Ci) (**Figure 3C**; **Supplementary Figure S1D**) increasing by 9.68% and 13.55% respectively at pH 6.5 compared to the control.

### 3.4 Effects of AMF on root function and reactive oxygen species and antioxidant enzymes under different pH gradients

**Figure 4:**
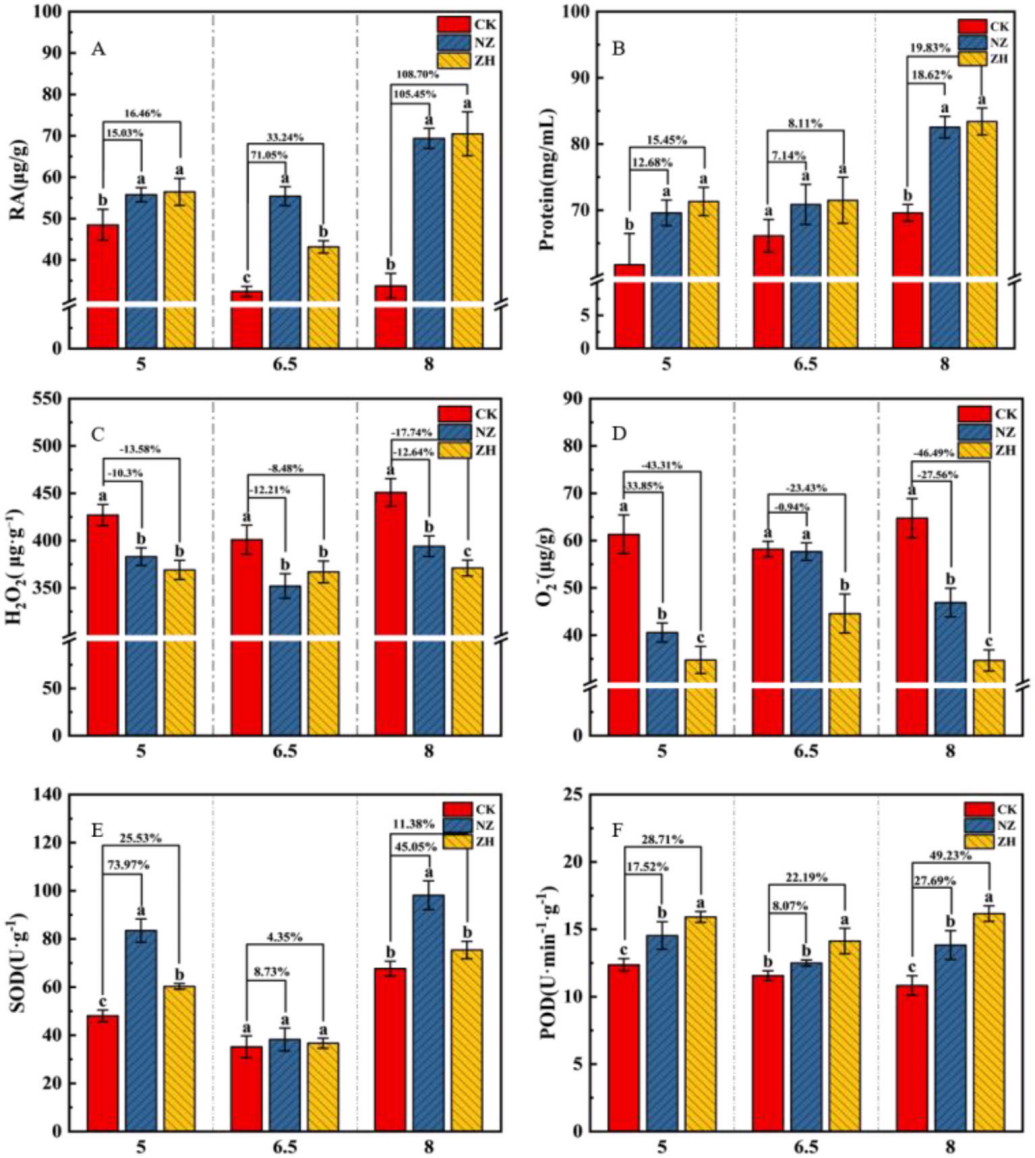
Effects of AMF on root function and reactive oxygen species and antioxidant enzymes under different pH gradients. Root vitality **(A)**, protein content **(B)**, hydrogen peroxide **(C)**, superoxide anion **(D)**, SOD superoxide dismutase **(E)**, peroxidase **(F)**. *S. viscosum*(NZ) and *G. chinensis*(ZH). The percentages indicate the enhancement compared to the CK treatment, with the NZ and ZH treatments showing respective percentage increases. Lowercase letters indicate significant differences between treatments (P < 0.05). Values shown are the mean ± standard error (n = 3).

Different pH gradients affect the physiological state of grapevine roots by altering root vitality, protein content, ROS, and antioxidant enzyme activity (**Figure 4**). Compared to pH 6.5, both pH 5 and pH 8 increased MDA levels in the roots, activating ROS and antioxidant enzyme activity in the roots (**Figure 4**; **Supplementary Figure S1G**). At pH 5, the protein content in the roots decreased, while at pH 8, it increased (**Figure 4B**). The addition of AMF reduced the accumulation of malondialdehyde and reactive oxygen species in plant roots, enhancing root vitality and antioxidant enzyme activity, thereby alleviating the damage caused by acid and base stress on plant roots. The treatment with ZH showed the best overall performance, where at pH 8, root vitality, protein content, and POD activity increased significantly by 108.7%, 19.83%, and 49.23% respectively compared to the control, while MDA, H_2_O_2_, and O_2_^-^ levels decreased by 35.79%, 17.74%, and 46.69% respectively (**Figures 4A, B, F, C, D** and **Supplementary Figure S1G**). The NZ treatment significantly increased SOD activity (**Figure 4E**), with a 73.97% increase compared to the control at pH 5. 

### 3.5 The impact of AMF on Fe absorption and transport under different pH gradients

**Figure 5:**
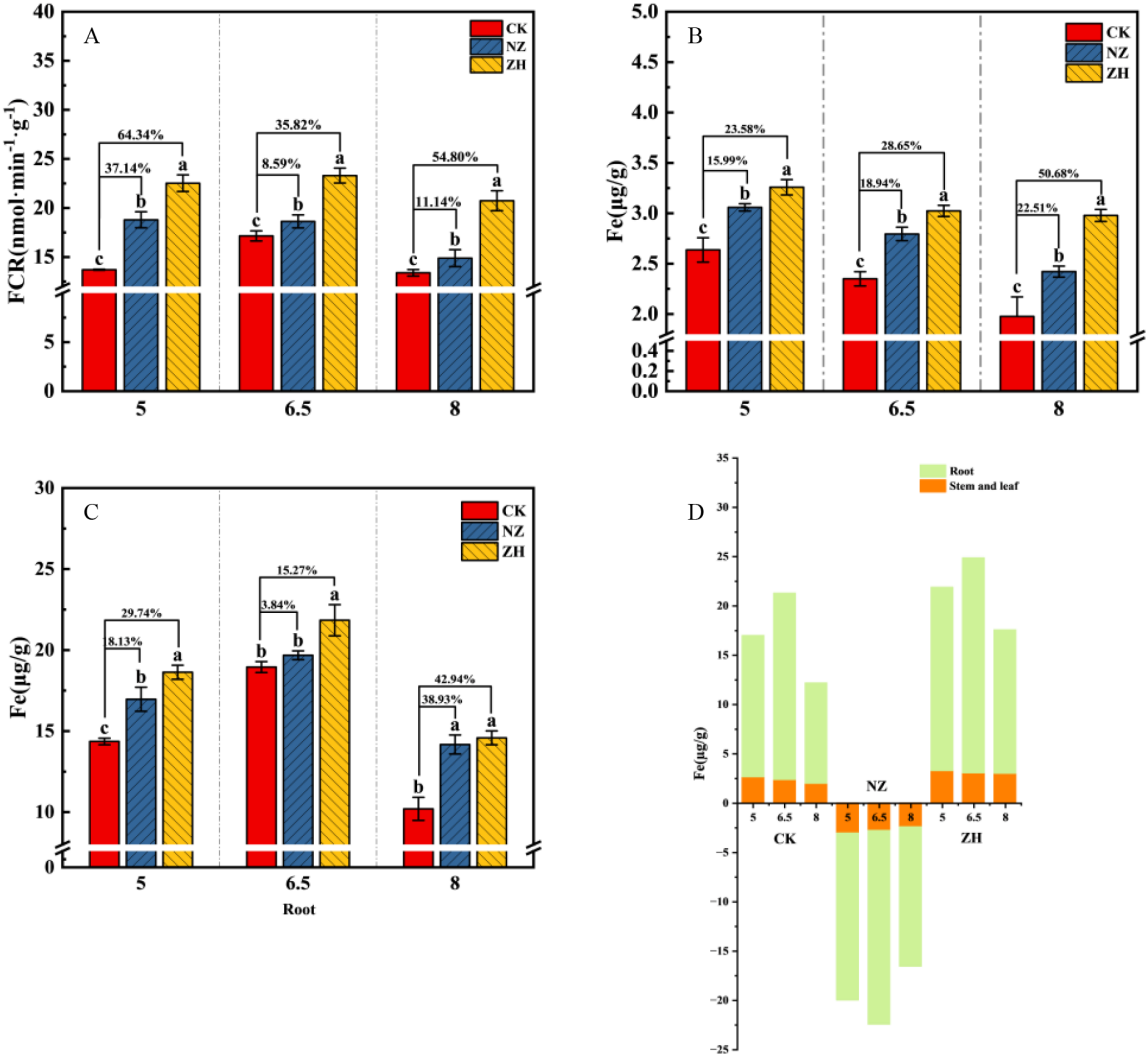
The impact of AMF on Fe absorption and transport under different pH gradients. Root FCR high-iron reductase **(A)**, shoot Fe **(B)**, root Fe **(C)**, plant Fe content **(D)**. *S. viscosum*(NZ) and *G. chinensis*(ZH). The percentages indicate the enhancement compared to the CK treatment, with the NZ and ZH treatments showing respective percentage increases. Lowercase letters indicate significant differences between treatments (P < 0.05). Values shown are the mean ± standard error (n = 3).

Different pH gradients influence the efficiency of iron absorption in plants by regulating the activity of ferrous reductase (FCR) (**Figure 5**). In the control treatment (CK), compared to pH 6.5, both pH 5 and pH 8 inhibited FCR enzyme activity (**Figure 5A**), with pH 8 significantly reducing the iron content in both the aerial and underground organs of the plants (**Figures 5B, C, D**). The addition of AMF greatly enhanced the FCR enzyme activity in the roots, significantly alleviating the impact of pH on iron absorption and transport (**Figure 5**). The ZH treatment exhibited the best overall performance; at pH 5, the FCR enzyme activity increased by 64.34% compared to the CK treatment (**Figure 5A**), while the iron content in the aerial and underground parts increased by 23.58% and 29.74%, respectively (**Figures 5B, C**). At pH 8, the FCR enzyme activity increased significantly by 54.8% (**Figure 5A**), and the iron content in the aerial and underground parts increased significantly by 50.68% and 42.94%, respectively (**Figures 5B, C**).

### 3.6 Effect of AMF on mineral elements in grapes under different pH gradients

**Figure 6:**
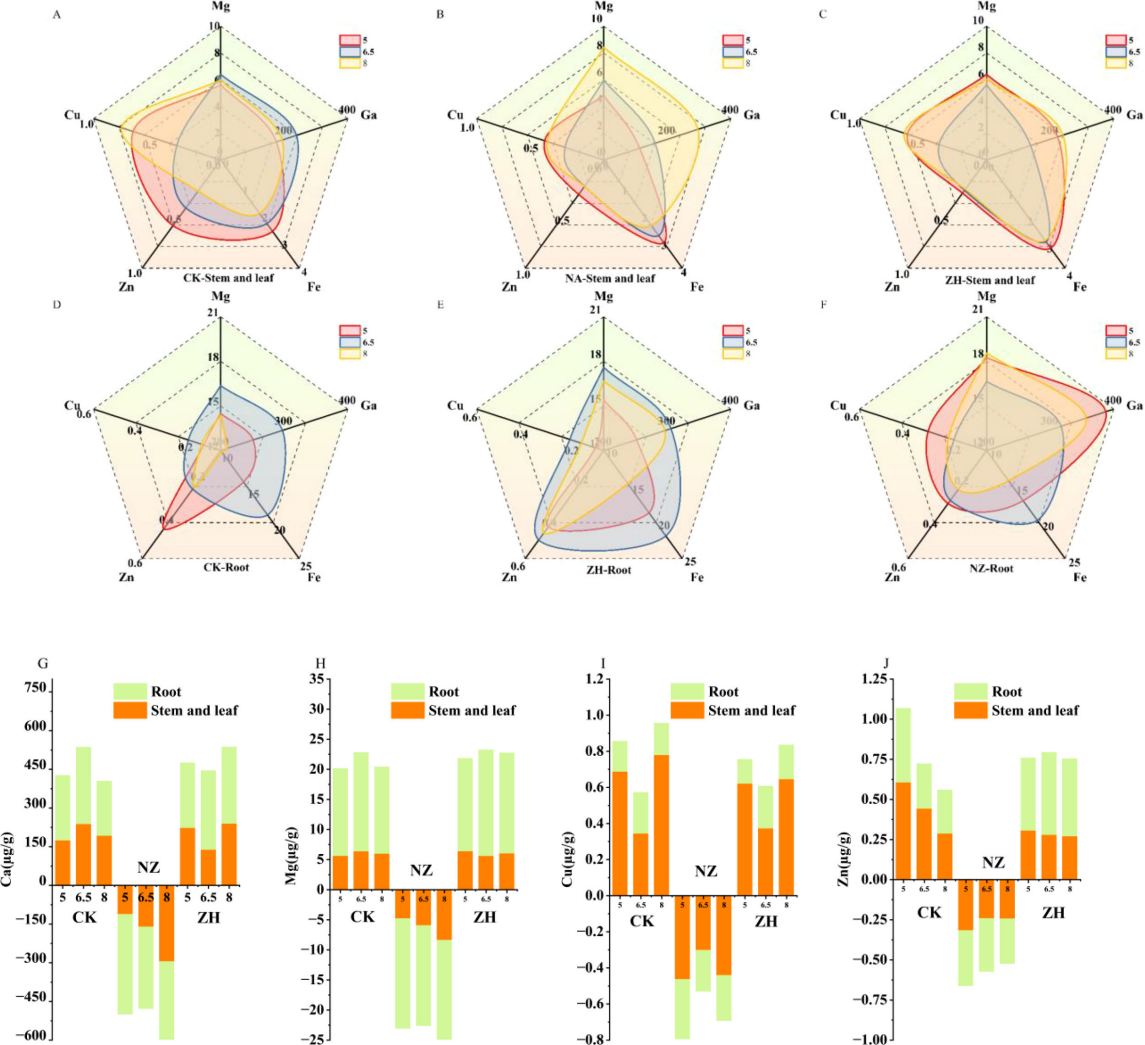
Effect of AMF on trace elements in grapes under different pH gradients. CK, NZ, ZH treatment aboveground part **(A–C)**, CK, NZ, ZH treatment underground part **(D–F)**, within the plant Mg, Ca, Cu, Zn **(G–J)**. *S. viscosum*(NZ) and *G. chinensis*(ZH).

The impact of different pH levels on the absorption and transport of Ca, Mg, Zn, and Cu by grapevine roots (**Figure 6**). Compared to pH 6.5, both pH 5 and pH 8 inhibit the Mg and Ca content in both aerial and underground organs (**Figures 6G, H**). AMF enhance nutrient utilization efficiency and alleviate metal stress by synergistically regulating the underground absorption and aerial redistribution of Ca, Mg, Zn, and Cu (**Figures 6A, B, C**). The addition of AMF significantly increased Mg and Ca content while reducing Zn and Cu content (**Figures 6B, C**). The NZ treatment showed the most significant improvement in trace element content; at pH=8, the Mg and Ca content in the aerial parts increased by 40.79% and 53.47%, respectively (**Supplementary Figure S2A, B**), while the underground Mg and Ca content increased by 28.98% and 69.26%, respectively (**Supplementary Figure S2E, F**), and the Cu content in the aerial parts significantly decreased by 43.16% (**Supplementary Figure S2C**). At pH 5, the underground Cu content significantly increased by 98% (**Supplementary Figure S2G**). The ZH treatment increased the Zn content in the roots, with an 84.43% increase in underground Zn content at pH 6.5 (**Supplementary Figure S2H**).

### 3.7 Correlation analysis between plant biomass and leaf and root functions, antioxidant systems, mineral elements

**Figure 7:**
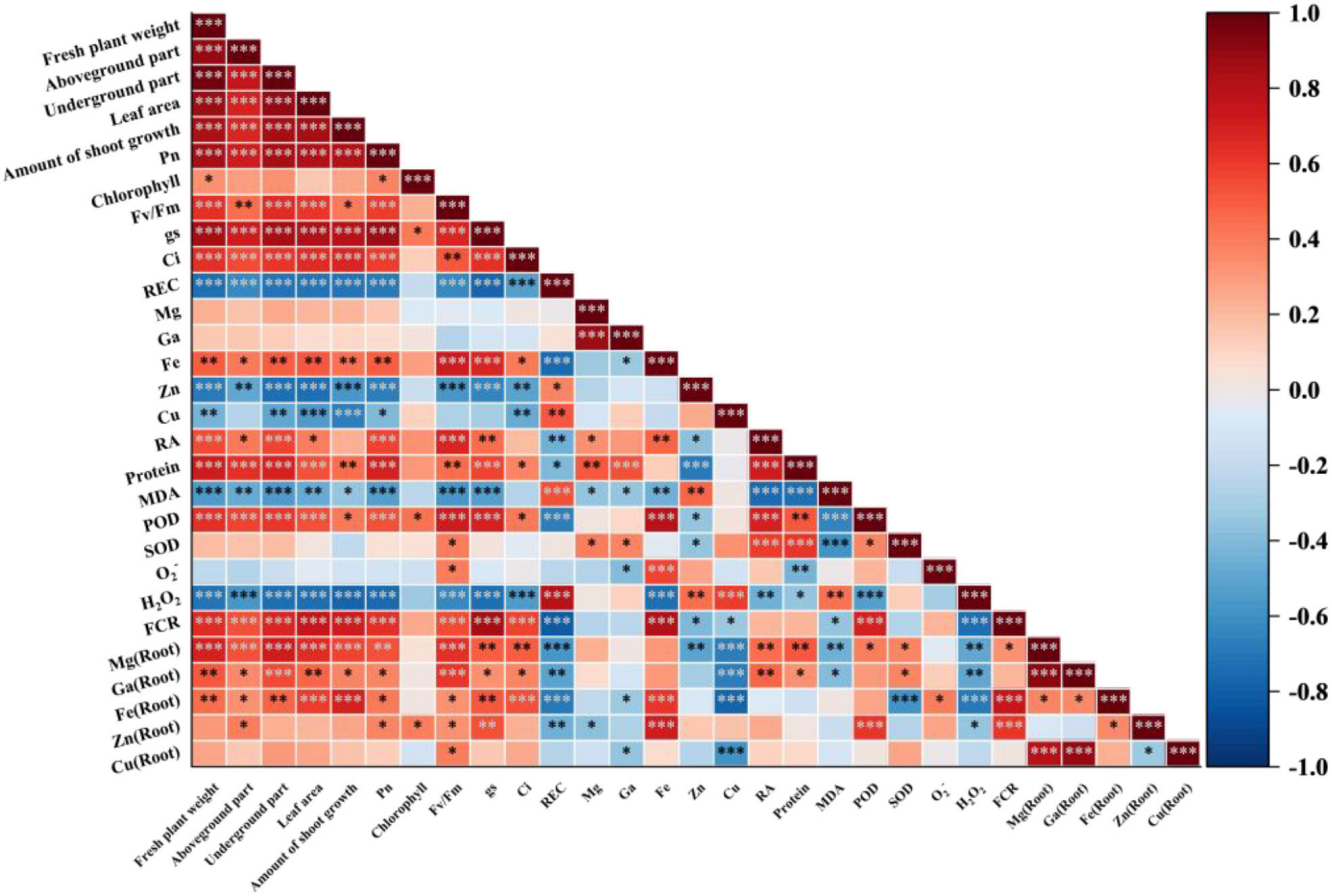
Pearson correlation analysis.

Through Pearson correlation analysis, the positive and negative correlations among various plant indicators are not influenced by pH and AMF (**Supplementary Figure S4**). There is a significant positive correlation between plant biomass, net photosynthetic rate, chlorophyll, fluorescence, intercellular carbon dioxide concentration, stomatal conductance, and root vitality. The indicators of plant biomass, net photosynthetic rate, chlorophyll, fluorescence, etc., show positive correlations with the aboveground Mg, Ca, Fe, and underground Mg, Ca, Fe, Zn, Cu; while they exhibit negative correlations with aboveground Zn and Cu, indicating that the inhibition of Zn and Cu transport upwards is beneficial for plant growth (**Figure 7**).

The aboveground Fe is negatively correlated with aboveground Mg, Ca, Zn, Cu, and positively correlated with underground Mg, Ca, Zn, Cu; underground Fe shows positive correlations with both aboveground and underground Mg, Ca, Zn, Cu. Aboveground Mg and Ca are negatively correlated with both aboveground and underground Zn, Cu. Underground Mg and Ca exhibit negative correlations with aboveground Zn, Cu and underground Zn, while showing positive correlations with underground Cu (**Figure 7**). In summary, the element Fe may play a key role in regulating Mg, Ca, Zn, and Cu elements and promoting grapevine growth.

### 3.8 Comparison of principal component and cluster analyses of grape physiological indicators across treatments

**Figure 8:**
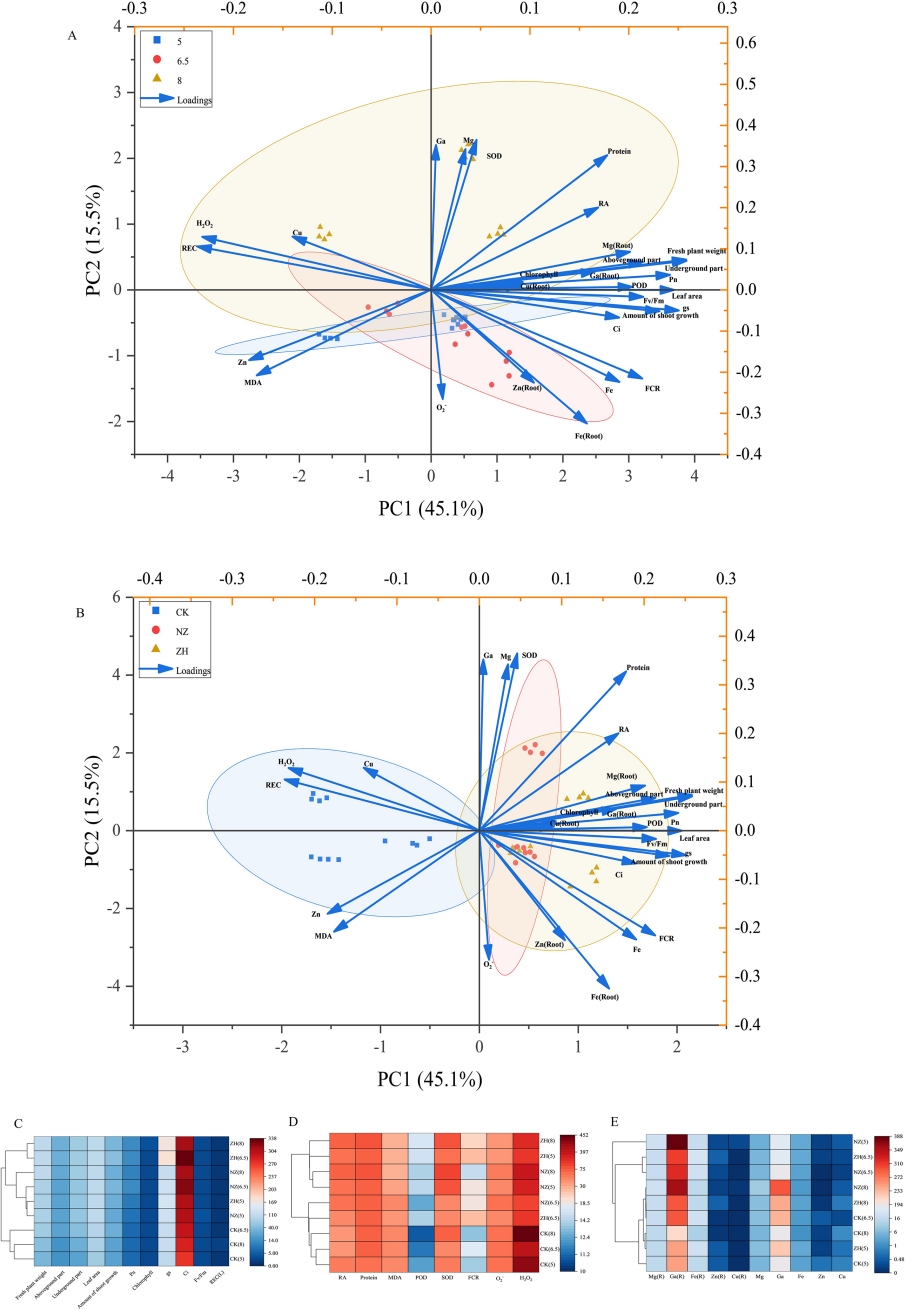
Principal Component Analysis. Principal component analysis of the environmental factors across different pH gradients **(A)**, principal component analysis of environmental factors across treatments **(B)**. Plant and leaf functions Heat Map with Dendrogram **(C)**, root system functions Heat Map with Dendrogram **(D)**, secondary and micronutrients Heat Map with Dendrogram **(E)**.

Combining principal component analysis with the Single Linkage clustering method, this study conducts a comprehensive analysis of the impact on plant growth and development through Cosine similarity. As illustrated in the figures, the effects of different pH levels on plant growth and development vary significantly, with pH 5 and pH 8 showing significant differences when compared to pH 6.5 (**Supplementary Figure S5**). In contrast to pH 6.5, pH 5 and pH 8 are relatively distant from each other (**Figure 8A**). Under the treatments of NZ and ZH, the differences among the three pH gradients are markedly reduced (**Supplementary Figure S5E, F**). There is a significant difference in the impact on plants between non-inoculated AMF and inoculated AMF (**Figure 8B**), with the effects of NZ and ZH treatments on plant growth and development being more pronounced. Notably, at pH 5 and pH 8, significant differences are observed among the three treatments (**Supplementary Figure S5B, C**), whereas at the pH 6.5 gradient, although differences exist among the three treatments, they are not significant (**Supplementary Figure S5**).

At pH 5, the effects of NZ treatment and ZH treatment on plant growth and development are similar (**Figure 8C**), while at pH 6.5 and pH 8, ZH treatment outperforms NZ treatment (**Figure 8C**). At pH 6.5, the effects on the absorption of trace elements in both the aerial and underground parts are comparable between NZ treatment and ZH treatment; however, at pH 5 and pH 8, NZ treatment is superior to ZH treatment (**Figure 8E**). In all three pH environments, ZH treatment has a more favorable impact on root function compared to NZ treatment (**Figure 8D**). Overall, the treatments without AMF show similar effects on plant growth and development under acid and base stress, while inoculation with AMF can effectively alleviate the impact of pH on plants. Among them, NZ treatment is more beneficial for the absorption and utilization of trace elements, whereas ZH treatment is more advantageous for alleviating acid and base stress and promoting growth.

## 4 Discussion

### 4.1 Constraints of different pH environments on the physiological functions of grapevine leaves and roots

Soil pH is a master regulator of rhizosphere conditions, nutrient bioavailability and plant physiological performance. pH−induced imbalances have become a major abiotic constraint to the sustainable development of viticulture. The use of an inert matrix (river sand and gravel) minimised confounding effects from soil organic matter and native microbes, allowing a more precise assessment of pH impacts. Grapevines grown at pH 6.5 (non−inoculated) attained the highest biomass, confirming that near−neutral pH provides optimal conditions for nutrient supply and physiological metabolism (Lisek and Popińska, 2025; Maru and Martin, 2004; Rezende et al., 2024). By contrast, both acidic (pH 5.0) and alkaline (pH 8.0) conditions directly impaired root and leaf structure, suppressed growth and hindered nutrient uptake. Relative to pH 6.5, both pH 5.0 and pH 8.0 markedly elevated root MDA and ROS levels and leaf REC, indicating that extreme rhizosphere pH causes peroxidative membrane damage and increased permeability, which in turn disrupts root uptake and leaf function (Helena et al., 2021; Jie et al., 2022; Zuo et al., 2025). These results align with earlier studies. Interestingly, at pH 6.5 we observed a decrease in leaf fluorescence and shoot Fe proportion, an increase in root Fe proportion, and a decline in root activity, despite maximal FCR activity and total plant Fe content. This indicates that the upward transport of Fe elements is relatively hindered at pH 6.5, potentially leading to a typical phenomenon of ‘functional iron deficiency.’ This phenomenon is often explained by the presence of a large amount of Fe in the root system, which cannot be effectively transported to the aboveground parts (especially the chloroplasts) for photosynthesis, resulting in functional iron deficiency in the leaves (López-Millán et al., 2001; Yanyan et al., 2023). It validates that the efficiency of nutrient absorption by the root system is an important physiological aspect regulating plant growth status, and its absorption and transport capacity directly depend on the synergistic effect between root physiological activity and the rhizosphere environment.

### 4.2 AMF alleviate pH stress by modulating root antioxidant defence

AMF are well known for their roles in stress mitigation and ecosystem restoration (Hu et al., 2022; Juge, 2025; Tang et al., 2023). In our study, AMF inoculation significantly improved grapevine performance across all pH treatments by enhancing leaf and root physiological activity, boosting stress tolerance and increasing nutrient translocation efficiency. On one hand, the infection formed a mycelial network that is more extensive than the root system, thereby expanding the radius of nutrient element absorption and increasing the protein content in the roots (Peng et al., 2025). On the other hand, it relies on the synergistic optimization of leaf photosynthesis, root vitality, and antioxidant enzyme activity. Although colonisation frequencies were somewhat reduced under acidic and alkaline stress relative to pH 6.5, AMF still significantly increased grapevine biomass at both extremes, indicating effective symbiosis even under suboptimal pH. AMF increases the infection frequency, expands the hyphal area, and enhances root vitality through improved metabolism and energy supply. The increase in root protein content can promote enzyme synthesis and reduce reactive oxygen species (Muzafar et al., 2023; Zhumei et al., 2022), which better protects root cell membranes and enzyme systems, improves root FCR activity and protein content (Kabir et al., 2020; Rahman et al., 2020), prevents protein degradation, withstands acid and base stress, and regulates the balance of mineral elements under different pH stresses in grapevines, thereby maintaining physiological homeostasis (Alao et al., 2024; Bingbing et al., 2020; Peng et al., 2024; Xu et al., 2024). These findings confirm the pivotal role of AMF in promoting plant growth and stress tolerance.

It is noteworthy that the efficiency of reactive oxygen species (O_2_^-^,H_2_O_2_) elimination in different pH environments is best achieved with *G. chinensis* (ZH) treatment, while the corresponding SOD content is highest with *S. viscosum* (NZ) treatment, which is inconsistent with previous studies (Peiman and Ewald, 2022). However, this study has certain limitations, as it only predicts that this may involve two distinctly different defense pathways. One pathway reduces the generation of reactive oxygen species (O_2_^-^,H_2_O_2_)directly from the source by inhibiting the electron transport chain of mitochondria or photosystem II, without the need to increase SOD content; the other pathway fails to completely prevent O_2_^-^ and H_2_O_2_ production, thereby activating SOD activity (Chang et al., 2023; Choudhury et al., 2025; Dongfeng et al., 2023; Feng et al., 2016; Gao et al., 2024; Yu et al., 2016). Although the SOD activity under *S. viscosum* (NZ) treatment is the highest, it also indicates that it has suffered a relatively larger oxidative shock, suggesting that the *G. chinensis* (ZH) treatment has a higher proactive role in prevention. These contrasting patterns highlight species−specific differences in ROS management and oxidative damage control, which ultimately contribute to the enhanced performance of AMF−colonised plants under pH stress (Cun et al., 2025; Mjokwe et al., 2025; Yakasai et al., 2025).

Soil pH can differentially affect AMF spore germination and hyphal growth; colonisation frequency is generally highest near pH 6.5. Following acid and base stress, the infection frequency of AMF in the root system decreases, disrupting the symbiotic relationship between the fungi and the roots (Reiermann et al., 2025). In this study, compared to acidic environments, the effects of AMF on biomass, leaf photosynthesis, root vitality, FCR enzyme activity, reactive oxygen species (ROS) metabolism, and the antioxidant system were significantly more pronounced in alkaline environments. This is related to the infection frequency and adaptability of AMF in alkaline conditions (Faria Jorge et al., 2022; Li et al., 2025; Mohammed et al., 2022; Weihong et al., 2023). FCR activity is particularly sensitive to high carbonate levels. AMF may alleviate carbonate stress by physically shielding roots with their hyphal network and by secreting organic acids that lower rhizosphere carbonate concentration, thereby preserving FCR function in alkaline soils. The higher colonisation frequency at pH 8 than at pH 5 resulted in denser hyphal networks, elevated root protein, enhanced FCR activity and improved Fe uptake and translocation, underscoring the strong pH−dependence of AMF−mediated growth promotion in grapevine.

### 4.3 AMF balance the distribution of mineral elements in grapevines

The five mineral elements Mg, Ca, Fe, Zn, and Cu are essential nutritional components for the growth and development of grapevines. Their processes of absorption, transport, and accumulation do not occur independently; rather, they form a close synergy and antagonism, which is also constrained by the pH environment of the rhizosphere (Cifizzari et al., 2023). Ca, as a core component of the cell wall, when insufficiently absorbed, exacerbates the erosion of root tip cells by H+ under acidic conditions, further decreasing nutrient absorption efficiency (Alonso et al., 2022; Sivakumar et al., 2022). Mg and Fe, which are important nutrients involved in photosynthesis, when reduced in content, inhibit the photosynthetic rate of leaves (Amorim, 2019; Bai et al., 2024; Li et al., 2024). Zn and Cu, as key components of plant antioxidant enzymes, when absorbed in an unbalanced manner, intensify the accumulation of reactive oxygen species in the roots under pH stress, further disrupting the stability of the root ion transport system and leading to impaired nutrient absorption synergy (Liu et al., 2024; Michael and Krishnaswamy, 2011; Tavallali et al., 2010).

Disruption of rhizosphere pH can trigger competitive uptake among mineral elements, upsetting the nutritional balance. This leads to suppressed vine growth, reduced shoot and root Mg and Ca concentrations, and altered Fe, Zn and Cu dynamics through changes in root physiology. In acidic soils, high H^+^ enhances Fe solubility, diminishing the need for FCR−mediated reduction and favouring Fe translocation to shoots (Barus et al., 2024; Máté et al., 2021; Mohamed et al., 2022). Simultaneously, ion competition impairs transmembrane nutrient transport, causing Ca and Mg to be retained in roots to protect root tips from acid damage (Bojórquez-Quintal et al., 2017; Kochian et al., 2004). In contrast, in alkaline environments, the effectiveness of Fe decreases, inducing compensatory activation of FCR (Mahender et al., 2019; Máté et al., 2021; Yanyan et al., 2023). In this study, At pH 6.5, high FCR activity facilitated efficient Fe uptake while sustaining root physiological function, thereby boosting root vigour and promoting the active uptake and accumulation of Mg, Ca, Zn and Cu, establishing a virtuous cycle of multiple element synergism (Kwakye et al., 2022). The higher root protein content at pH 8 compared to pH 5, together with the maintenance of FCR activity, further supports this interpretation (Clúa et al., 2024).

AMF alleviate H^+^ and carbonate stress by developing extensive hyphal networks, secreting organic acids and enzymes, and modulating the rhizosphere microenvironment. They improve root physiology, increase mineral element solubility and promote uptake and translocation, thereby enhancing the acquisition of Ca, Mg and Fe. Moreover, AMF secrete metabolites that balance Cu and Zn uptake, preventing excessive heavy metal accumulation (Naheeda et al., 2019). In this study, *S. viscosum* and *G. chinensis* enhanced the uptake and shoot translocation of Ca, Mg and Fe, supporting shoot growth. However, they restricted upward transport of Cu and Zn, retaining these metals in roots and thus preventing their accumulation in leaves. This differential regulation not only optimizes the supply of essential mineral elements but also reduces the potential accumulation of heavy metals in the aerial parts, reflecting the synergistic regulatory role of AMF in plant mineral nutrition and heavy metal detoxification. There are also differences in effectiveness among different AMF. The treatment with *S. viscosum* (NZ) in acidic environments enhanced the fixation of Mg and Ca elements at the root level while suppressing their upward transport. Conversely, the treatment with *G. chinensis* (ZH) in acidic environments promoted the upward transport of Ca, Mg, Fe, and Cu. However, there are currently no precise research reports that clearly distinguish the functional differences between *S. viscosum* and *G. chinensis*. The adaptation to acidic and alkaline environments can only be inferred through infection frequency and the resulting outcomes.

## 5 Conclusion

This study demonstrates that soil pH and arbuscular mycorrhizal fungi (AMF) species interactively influence grapevine growth, development and mineral element dynamics. We confirm synergistic and antagonistic interactions among Fe, Mg, Ca, Zn and Cu in grapevines. Under alkaline stress (pH 8), grapevines exhibited higher biomass, photosynthesis and root vigour than under acidic stress (pH 5), favouring root retention and shoot translocation of Mg, Ca and Cu. Conversely, acidic conditions (pH 5) were more conducive to Fe and Zn uptake and shoot translocation. Overall, both types of AMF perform better in alkaline environments, with *S. viscosum* being more beneficial for the absorption and transport of Mg, Ca, Fe, Zn, and Cu within grapevines. In contrast, *G. chinensis* significantly enhanced the functions of leaves and roots, markedly increased FCR enzyme activity and protein content, and strengthened the absorption and utilization of Fe while improving the fixation of Zn by the roots. These findings provide a basis for selecting AMF strains tailored to specific soil pH conditions in viticulture, enabling precision matching of fungal partners to vine nutritional requirements. This will improve the efficiency of applications based on AMF, enhance grapevine tolerance to pH stress and support sustainable production of high quality.

## Data Availability

The original contributions presented in the study are included in the article/**Supplementary Material**. Further inquiries can be directed to the corresponding authors.
