# Supplementary material for: Arbuscular mycorrhizal fungi regulate mineral element distribution in grapevines under pH stress
Source: Front Plant Sci. 2026 Apr 7;17:1785602. doi: 10.3389/fpls.2026.1785602 (PMC13096024; doi:10.3389/fpls.2026.1785602)
Supplement: Supplementary file 1 [file DataSheet1.docx]

| **Hoglan Nutrient Solution** | | | |
| --- | --- | --- | --- |
| **Category(A)** | **Reagent(B)** | **Concentration(C)** | **Volume(D)** |
| **1** | KNO_3_ | 101g/L | 5ml |
| **2** | KH_2_PO_4_ | 136 g/L | 1 ml |
| **3** | Ca(NO_3_)_2_ | 236 g/L | 5 ml |
| **4** | MgSO_4_ | 246.5 g/L | 2 ml |
| **5** | H_3_BO_3_ | 2.86 g/L | 1 ml |
|  | MnCl_2_·4H_2_O | 1.81 g/L | 1 ml |
|  | ZnSO_4_·7H_2_O | 0.22 g/L | 1 ml |
|  | CuSO_4_·5H_2_O | 0.08 g/L | 1 ml |
| **6** | EDTA-Fe | 10μmol /L | 5 ml |

Table. S1: Hoglan Nutrient Solution. B and C are prepared with sterile water, stored according to A, protected from light, and finally mixed in 1 L according to D for immediate use.

| **Arbuscular Mycorrhizal Fungi** | **pH** | **Infection frequency(%)** |
| --- | --- | --- |
| ***Septoglomus viscosum*** | 5 | 48 |
|  | 6.5 | 68 |
|  | 8 | 57 |
| ***Glomus chinensis*** | 5 | 51 |
|  | 6.5 | 70 |
|  | 8 | 63 |

Table. S2: The infection frequency of Septoglomus viscosum and Glomus chinensis was determined at different pH levels.

Fig. S1: The effects of AMF on leaves under varying pH conditions. Chlorophyll a (A)、Cxcb(B)、C (C)、Chlorophyll b (D)、Ci(E)、gs (F)、root MDA (G)

Fig. S2: The influence of AMF on Mg, Ca, Cu, and Zn levels in the aboveground parts (A, B, C, D) and underground parts (E, F, G, H) across varying pH conditions.





Fig.S 3: The variations of Fe, Mg, Ca, Cu, and Zn among the CK, NZ, and ZH treatments under different pH environments (Total).aboveground parts (A), underground parts (B).

Fig. S4: Pearson correlation analysis of grape plant indicators across different groups. pH=5(A)、pH=6.5(B)、pH=8(C)、CK(D)、NZ(E)、ZH(F).

Fig. S5: Principal component analysis of grape traits across different groups. pH=5(A)、pH=6.5(B)、pH=8(C)、CK(D)、NZ(E)、ZH(F).
